# Urinary Concentrations of Insecticide and Herbicide Metabolites among Pregnant Women in Rural Ghana: A Pilot Study

**DOI:** 10.3390/ijerph14040354

**Published:** 2017-03-29

**Authors:** Blair J. Wylie, Kenneth A. Ae-Ngibise, Ellen A. Boamah, Mohammed Mujtaba, Carmen Messerlian, Russ Hauser, Brent Coull, Antonia M. Calafat, Darby Jack, Patrick L. Kinney, Robin Whyatt, Seth Owusu-Agyei, Kwaku P. Asante

**Affiliations:** 1 Division of Maternal-Fetal Medicine, Vincent Department of Obstetrics and Gynecology, Massachusetts General Hospital and Harvard Medical School, 55 Fruit Street, Boston, MA 02114, USA; 2 Department of Environmental Health, Harvard T.H. Chan School of Public Health, 677 Huntington Avenue, Boston, MA 02115, USA; cmesser@hsph.harvard.edu (C.M.); rhauser@hsph.harvard.edu (R.H.); bcoull@hsph.harvard.edu (B.C.); 3 Kintampo Health Research Centre, Ghana Health Service, P.O. Box 200, Brong Ahafo Region, Kintampo 00233, Ghana; kenneth.asayah@kintampo-hrc.org (K.A.A.-N.); ellen.boamah@kintampo-hrc.org (E.A.B.); mohammed.mujtaba@kintampo-hrc.org (M.M.); seth.owusu-agyei@kintampo-hrc.org (S.O.-A.); kwakupoku.asante@kintampo-hrc.org (K.P.A.); 4 Department of Statistics, Harvard T.H. Chan School of Public Health, 677 Huntington Avenue, Boston, MA 02115, USA; 5 Centers for Disease Control and Prevention, 4770 Buford Hwy, Atlanta, GA 30341, USA; aic7@cdc.gov; 6 Department of Environmental Health Sciences, Mailman School of Public Health, Columbia University, 722 West 168th Street, New York, NY 10032, USA; dj2183@columbia.edu (D.J.); plk3@columbia.edu (P.L.K.); rmw5@cumc.columbia.edu (R.W.)

**Keywords:** insecticides, herbicides, pregnancy, organophosphates, pyrethroids

## Abstract

Use of pesticides by households in rural Ghana is common for residential pest control, agricultural use, and for the reduction of vectors carrying disease. However, few data are available about exposure to pesticides among this population. Our objective was to quantify urinary concentrations of metabolites of organophosphate (OP), pyrethroid, and select herbicides during pregnancy, and to explore exposure determinants. In 2014, 17 pregnant women from rural Ghana were surveyed about household pesticide use and provided weekly first morning urine voids during three visits (*n* = 51 samples). A total of 90.1% (46/51) of samples had detectable OP metabolites [geometric mean, GM (95% CI): 3,5,6-trichloro-2-pyridinol 0.54 µg/L (0.36–0.81), para-nitrophenol 0.71 µg/L (0.51–1.00)], 75.5% (37/49) had detectable pyrethroid metabolites [GM: 3-phenoxybenzoic acid 0.23 µg/L (0.17, 0.32)], and 70.5% (36/51) had detectable 2,4-dichlorophenoxyacetic acid levels, a herbicide [GM: 0.46 µg/L (0.29–0.73)]. Concentrations of para-nitrophenol and 2,4-dichlorophenoxyacetic acid in Ghanaian pregnant women appear higher when compared to nonpregnant reproductive-aged women in a reference U.S. population. Larger studies are necessary to more fully explore predictors of exposure in this population.

## 1. Introduction

Prenatal exposure to nonpersistent pesticides has been linked to adverse impacts on neurodevelopment, particularly for organophosphates, but also for pyrethroids [1,2,3,4,5]. Little is known about the exposure to these nonpersistent pesticides among African populations [6]. It is particularly important to fill this knowledge gap for pregnant women, because of fetuses’ vulnerability to environmental toxins given their rapid development and the immaturity of their metabolic pathways [7].

A variety of pesticides are readily available for purchase in Ghana [8,9]. The promotion of pesticide benefits by chemical companies, an inadequate regulatory environment, and a limited understanding of the potential dangers among users, may contribute to the misuse of these chemicals by African households and farms. These factors, in turn, may increase human exposure to these chemicals [10,11]. Pesticide exposure among Ghanaians may also occur through the use of insecticides for vector control against malaria and other insect-borne diseases [12]. For malaria control, insecticide-treated bednets are now standard and pyrethroids are the most common insecticide used for the treatment of nets. Additionally, the Ghanaian government performs indoor residual spraying with insecticides in some communities, to reduce malaria [13]. Many households also purchase insecticides to minimize the nuisance of pests in and around the home.

In the Brong Ahafo Region of Ghana, we have previously reported that 71.5% of the households surveyed used pesticides (1040/1455) [14]. This included herbicides (68.9% of households), insecticides (61.0% of households), and rodenticides (44.5% of households). Hazardous practices identified in the area included self-reported storage in the home in close proximity to food, the re-use of empty insecticide containers for food storage, and inappropriate application just before harvest. Furthermore, only a minority of people reported the use of protective clothing such as gloves or masks during pesticide applications (301/1040, 28.9%) [14]. Reports describing frequent pesticide use among households and a lack of personal protection have been published from other African regions [15,16]. Whether these hazardous practices result in higher levels of exposure remains unknown.

The primary objective of this study was to assess the exposure to insecticides and herbicides among pregnant women in rural Ghana. Secondarily, we aimed to explore whether exposures were associated with farming practices, housing conditions, or the use of insecticide-treated bednets, in order to identify potential predictors for confirmation in larger studies.

## 2. Material and Methods

### 2.1. Study Area

The study was conducted in the Brong Ahafo Region of Ghana. The study area is predominantly rural, and subsistence farming is the main economic activity [17]. Malaria remains the primary reason for outpatient visits to health facilities in the region. A variety of shops sell pesticides for household and agricultural pest control.

### 2.2. Participant Selection

We utilized the Ghana Randomized Air Pollution and Health Study (GRAPHS; Trial Registration NCT01335490) to recruit pregnant women for this pilot study on prenatal pesticide exposure. GRAPHS was undertaken in 35 community clusters by the Kintampo Health Research Centre (KHRC) in Brong Ahafo, to understand whether the use of improved cook stoves or fuels during pregnancy can improve birth weight and reduce pneumonia during the first year of life [18]. A simple random sampling method was used to select women from a list of the 1090 GRAPHS participants under active surveillance as of September 2014. Selected women were approached for participation in this pesticide exposure study and were provided with information about the additional study procedures required, including the answering of a structured questionnaire on household pesticide use and the provision of three urine sample collections.

### 2.3. Ethical Considerations

Written informed consent was obtained for all who agreed to participate in the study. Women unable to provide consent by signature provided their thumb print. The thumb print was accompanied by the signature of a witness who was also present for the consenting process and was not part of the research team. Ethical approval was obtained from Partners Healthcare (overseeing Massachusetts General Hospital, Protocol #2015P001067), the KHRC Scientific Review Committee and Institutional Ethics Committee (Protocol #2014-17). The involvement of the Centers for Disease Control and Prevention (CDC) laboratory did not constitute engagement in human subjects’ research.

### 2.4. Data Sources

#### 2.4.1. Questionnaire Interview

A questionnaire was administered to participants about the household use of pesticides, personal and family agricultural activities, bednet use, and other antimalarial prevention strategies. Permission was obtained to access information from GRAPHS regarding demographics, socioeconomic factors, and other relevant household characteristics.

#### 2.4.2. Urine Sample Collection and Analysis

A total of three first morning urine voids were collected from participants in polypropylene specimen containers over three consecutive weekly home visits during pregnancy, following the study staff’s instructions. Samples were transported in coolers with ice packs on the same day of collection by GRAPHS Field Supervisors from the participants’ home to the KHRC Clinical Laboratory facility, and were stored at −80 °C in 10 mL polypropylene cryovials. Frozen samples were then shipped from Ghana in containers with dry ice to the CDC in Atlanta, Georgia, and stored at −80 °C until analysis.

Urine samples were analyzed at the CDC for three specific metabolites of organophosphate insecticides: 3,5,6-trichloro-2-pyridinol (TCPy, a metabolite of chlorpyrifos and chlorpyrifos methyl), 2-isopropyl-4-methyl-6-hydroxypyrimidine (IMPY, metabolite of diazinon), and para-nitrophenol (PNP, metabolite of methyl and ethyl parathion), and five nonspecific metabolites of organophosphate insecticides: diethyldithiophosphate (DEDTP), diethylphosphate (DEP), dimethyldithiophosphate (DMDTP), dimethylthiophosphate (DMTP), and diethylthiophophate (DETP). Samples were also analyzed for specific and non-specific metabolites of synthetic pyrethroid insecticides: 4-fluoro-3-phenoxybenzoic acid (4-F-3-PBA, metabolite of cyfluthrin), 3-phenoxybenzoic acid (3-PBA, metabolite of cyhalothrin, cypermethrin, deltamethrin, fenpropathrin, permethrin, and tralomethrin), and trans-3-(2,2-dichlorovinyl)-2,2-dimethylcyclopropane carboxylic acid (*trans*-DCCA, metabolite of permethrin, cypermethrin, cyfluthrin), as well as for two herbicide biomarkers: 2,4-dichlorophenoxyacetic acid (2,4-D), and 2,4,5-trichlorophenoxyacetic acid (2,4,5-T). We chose not to measure dichloro-diphenyl-trichloroethane (DDT) as this would require blood sampling and exposure has been previously documented among pregnant and lactating women in Africa [19,20,21,22]. Analyses were conducted using a modification of the solid phase extraction high performance liquid chromatography-isotope dilution tandem mass spectrometry approaches previously described [23,24]. Quality control was established by the repeat analysis of two in-house pools, whose target values and confidence limits were previously defined. Urine pesticide metabolite concentrations were determined both before and after the adjustment for urine dilution. Unadjusted concentrations were reported in µg analyte per L urine. Adjusted concentrations were reported in µg analyte per gram creatinine by multiplying the metabolite concentration (in µg/L) by [(1/creatinine in mg/dL) × (1000 mg/g) × (1 L/10 dL)] [25]. We calculated the percentage of pesticide biomarker concentrations above the limit of detection (LOD) and the range of detectable concentrations. Concentrations below the LOD were assigned the limit of detection divided by the square root of two [26].

### 2.5. Statistical Analyses

The metabolite concentrations were natural log-transformed to normalize the distribution and were used to estimate the geometric mean of the three urine samples per participant. Additional descriptive statistics, including the interquartile range and the 95th percentile, were then generated for each metabolite, both before and after the adjustment for creatinine. Descriptive statistics were generated for both creatinine-adjusted and unadjusted concentrations. A mixed-effects model with a random intercept was used to calculate the intraclass correlation coefficient (ICC) and determine the proportion of variance in metabolite concentrations attributable to within-person vs. between-person variability.

We then explored potential predictors of herbicide, pyrethroid, and organophosphate biomarker concentrations, by presenting geometric mean metabolite concentrations stratified by the predictor of interest (e.g., agricultural occupation, yes vs. no). For herbicides, pyrethroids, and organophosphate biomarkers, we considered agricultural practices and sources of household water as potential predictors. For pyrethroid metabolites, we additionally explored the the use of malaria prevention measures as potential exposure predictors. Given the small sample size, formal statistical testing was not performed.

Statistical analyses were conducted using SAS 9.4 (Research Triangle Institute, Cary, NC, USA).

## 3. Results

Between September and October of 2014, of the 17 women selected by simple random sampling of active GRAPHS participants, all (100%) agreed to participate and provided written informed consent. All were currently in their second or third trimester of pregnancy at enrollment. Demographics of the participants are presented in Table 1. The mean (SD) age of the participants was 26.2 years (±7.8) and most had been pregnant at least once before (14/17, 82.4%; mean gravidity 3.4 ± 2.8). Approximately half of the women (9/17, 52.9%) worked outside the home as a farmer, domestic worker, or in a salaried occupation (seamstress, hairdresser).

There was a range of pesticide use, both in the home and in the farm or garden (Table 1). The majority of women (14/17, 82.4%) used pesticides in their farm or garden. All women reported using herbicides in their household and approximately one third applied chemicals inside or around the home to kill insects or rodents (8/17, 35.2%). The most common locations for application of pesticide chemicals in the home were the sleeping areas (8/8, 100%), followed by the sitting room and kitchen (2/8, 25.0% for both). Among households using pesticide chemicals, women frequently participated in the spraying of pesticides (13/14, 92.9%), although this task declined during pregnancy (4/13, 30.8%). About half of the women reported carrying their children with them during spraying (6/13, 46.2%). Personal protective gear was rarely used during pesticide applications. Gloves were utilized by three women, masks by two, and protective clothing worn during spraying was employed by six women. Pesticides were typically purchased in the open market and only rarely from an accredited vendor.

Thirteen of the seventeen households (76.5%) owned bednets, and 64.7% of participants (11/17) reported sleeping under a bednet most nights. No participating households had been sprayed recently as part of the government’s antimalarial campaigns.

The 17 participants provided three first morning void urine samples each, producing a total of 51 samples. The samples were collected from each participant on a weekly basis, over 14 days.

### 3.1. Pesticide Exposure

**Figure 1:**
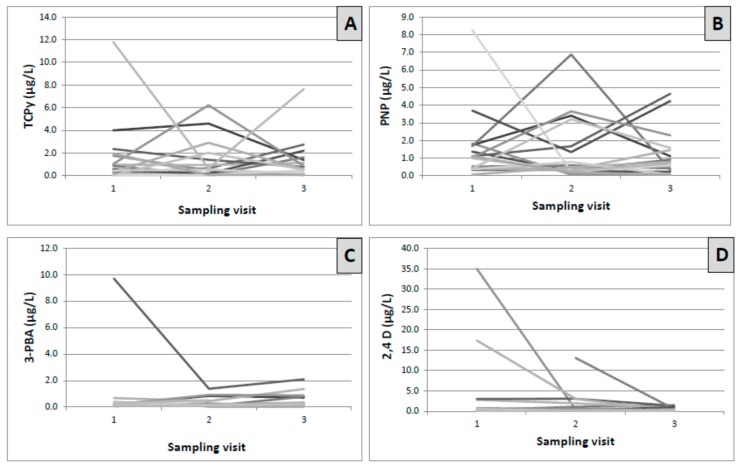
Variability of insecticide and herbicide metabolite concentrations at three sampling visits among 17 pregnant women in rural Ghana. Each line (different shades of gray) represents an individual pregnant woman. Legend: Creatinine-corrected metabolite concentrations at three successive sampling visits plotted per individual. (**A**) 3,5,6-Trichloro-2-pyridinol (TCPy) in µg/L, intraclass correlation coefficient (ICC) = 0.33; (**B**) *para*-Nitrophenol (PNP) in µg/L, ICC = 0.0; (**C**) 3-phenoxybenzoic acid (3-PBA) in µg/L, ICC = 0.31; and (**D**) 2,4-Dichlorophenoxyacetic acid (2,4-D) in µg/L, ICC = 0.06.

Summary results of insecticide and herbicide metabolite concentrations are presented in Table 2. The variability of metabolite concentrations over time within a single participant and between participants is visualized in Figure 1. We chose to present unadjusted metabolite concentrations after reviewing the distribution of creatinine concentrations and noting that urinary creatinine was <20 mg/dL in 11 of 51 samples (21.5%), which could lead to falsely elevated adjusted pesticide concentrations. Creatinine-adjusted values are presented for comparison in the supplement (See Supplementary Materials, Table S1).

#### 3.1.1. Organophosphate Insecticide Exposure

Specific organophosphate insecticide metabolites were frequently detected in our study population. Over 75% of the 51 samples had detectable TCPy (40/51, 78.4%) (Table 2). PNP was detected even more frequently (46/51, 90.1%). IMPY was the least frequently detected specific organophosphate biomarker (19/49, 38.8%). The nonspecific dialkyl phosphate (DAP) metabolites of organophosphate insecticides were infrequently detected (less than 25% of samples, Table 2). We then compared the samples with nondetectable DAP metabolites with concentrations of the two frequently detected specific OP metabolites, TCPy and PNP, and the following pattern emerged. The probability of a sample having undetectable DAPs was higher among samples with TCPy and PNP concentrations in the lower two quartiles, compared with those in the upper quartiles, although formal statistical testing was not performed secondary to sample size (data not shown).

The geometric mean (GM) concentration of TCPy was 0.54 µg/L (95% CI: 0.36, 0.81), with detectable measurements ranging from 0.11 to 11.8 µg/L (Table 2). The 95th percentile was 6.22 µg/L. For PNP, the GM concentration was 0.71 µg/L (95% CI: 0.51, 1.00), with detectable measurements ranging from 0.19 to 8.26 µg/L. The 95th percentile for PNP was 4.65 µg/L (Table 2). Geometric mean concentrations were not calculated for IMPY as the detection frequency was <50%. Most of the variance in the measurements was accounted for by within-subject variability for both TCPy and PNP (ICCs of 0.33 and 0.07, respectively) (Table 2 and Figure 1A,B).

#### 3.1.2. Pyrethroid Insecticide Exposure

The non-specific pyrethroid metabolite 3-PBA was the pyrethroid biomarker most commonly detected (37/49, 75.5%) (Table 2). 4-F-3-PBA was detected in less than 5% of samples (2/51, 3.9%). Similarly, *trans*-DCCA was detected in <5% of samples (2/51, 3.9%). The geometric mean 3-PBA concentration was 0.23 µg/L (95% CI: 0.17, 0.32), with a range of detectable measurements from 0.11 to 9.70 µg/L. The 95th percentile was 1.38 µg/L (Table 2). As with TCPy and PNP, within-subject variability accounted for most of the variance in 3-PBA concentrations (ICC of 0.31) (Table 2 and Figure 1C).

#### 3.1.3. Herbicide Exposure

2,4,5-T was not detected in any of the samples. In contrast, 2,4-D was frequently detected (36/51 samples, 70.6%) (Table 2). The geometric mean 2,4-D concentration was 0.46 µg/L (95% CI of 0.29, 0.73) and the 95th percentile was 17.4 µg/L. Detectable concentrations ranged from 0.15 to 166.0 µg/L. Within-subject variability accounted for 94% of the variance in 2,4-D concentrations (ICC of 0.06) (Table 2 and Figure 1D).

### 3.2. Exploring Associations of Insecticide and Herbicide Exposure with Farming Practices, Water Source, and Use of Bednets

We present the geometric mean metabolite concentration for TCPy, PNP, 3-PBA, or 2,4-D by the exposure category considered (e.g., ownership of bednet vs. not), but did not conduct formal statistical testing given the small sample size. However, a few suggestive patterns emerged. The source of water may be a predictor of higher urinary concentrations of 2,4-D, PNP and 3-PBA. Geometric mean 2,4-D concentrations were higher among women whose water was obtained from natural sources such as streams, rivers, or lakes, compared to women whose water was pumped, piped, or obtained from a public tap (1.57 µg/L vs. 0.31 µg/L) (Supplemental material, Table S2). Participants from households that relied on natural sources of water compared to women who used pumped, piped, or tap water, also appeared to have higher geometric mean PNP (0.96 µg/L vs. 0.65 µg/L) and 3-PBA (0.53 µg/L vs. 0.18 µg/L) concentrations. 2,4-D urinary concentrations also appeared higher among women who farmed as an occupation (0.95 µg/L vs. 0.31 µg/L) (Supplementary Materials, Table S2).

As only pyrethroid insecticides are approved for use to treat bednets in Ghana, we explored whether 3-PBA concentrations, a pyrethroid metabolite detected in many of our participants, varied by ownership and utilization of bednets. The mean 3-PBA concentrations appeared similar among those whose households owned a bednet, those who slept under a bednet most nights, or those who slept under a bednet the night prior to sampling, compared to those who did not.

## 4. Discussion

In this study, we demonstrated widespread pesticide exposure from the frequent detection of urinary concentrations of metabolites of organophosphate and pyrethroid insecticides, as well as select herbicides, in a sample of 17 pregnant women residing in an agricultural setting in Ghana, West Africa. Over 90% of urine samples had detectable organophosphate metabolites, over 75% had detectable pyrethroid metabolites, and over 70% had detectable 2,4-D levels, a herbicide.

### 4.1. Exposure Assessment

We present our pesticide exposure distributions from this Ghanaian population alongside the most recently published geometric means (in µg/L) and 95th percentiles among reproductive-aged females from the United States National Health and Nutrition Examination Survey (NHANES) for 2007–2008. NHANES was chosen as a reference as we utilized the same laboratory (CDC) to measure concentrations. An equivalent African reference or global standard does not currently exist [27]. Urinary concentrations during pregnancy may underestimate exposure secondary to the increased volume of distribution, as has been demonstrated for organophosphate pesticide exposure among an agricultural population in the U.S. [28]. That said, pregnancy specific references are not reported in NHANES and data from individual studies of pregnant women are difficult to compare with ours on account of differences in exposure measurements (e.g., air sampling). For organophosphates in particular, studies among pregnant U.S. females have typically reported nonspecific DAP metabolite concentrations [29,30,31,32]. As outlined in Section 4.2, DAP concentrations were unusually low among our participants in comparison to specific organophosphate metabolites, which we suspect may be related to field conditions precluding a direct comparison with a pregnant counterpart.

For PNP, the most frequently detected organophosphate metabolite in our samples, the geometric mean was higher for Ghanaian pregnant women than for reproductive-aged women in NHANES (0.71 µg/L vs. 0.40 µg/L), as was the 95th percentile (4.65 µg/L vs. 3.07 µg/L). In contrast, for the organophosphate TCPy, the geometric mean from our sample was lower than the geometric mean of the NHANES reproductive-aged females (0.54 µg/L vs. 0.71 µg/L), but the 95th percentile was higher (6.22 µg/L vs. 4.40 µg/L). For 3-PBA, the most frequently detected pyrethroid biomarker among the study samples, the geometric mean was lower than NHANES (0.23 µg/L vs. 0.42 µg/L), as was the 95th percentile (1.38 µg/L vs. 6.50 µg/L). For the herbicide 2,4-D, the geometric mean for our samples was 0.46 µg/L vs. 0.28 µg/L in NHANES, but the 95th percentile for participants was over 15 times higher, presenting a figure of 17.4 µg/L compared to 1.91 µg/L for NHANES.

In summary, in this study of pregnant women from a rural area of Ghana, urinary concentrations of biomarkers of select organophosphate insecticides (PNP) and of herbicides (2,4-D) appear higher than a nonpregnant female reference population in the US. Given the increased volume of distribution in pregnancy [28], the differences we observed for our Ghanaian participants compared to a nonpregnant reference group may have been even greater, if data were available from a pregnant referent group. We hypothesize that pyrethroid exposure may be lower among this group of Ghanaian pregnant women than in the U.S. general population, secondary to a ban in the early 2000s in the United States of the residential use of organophosphates and the consequent increase in pyrethroid use [33].

We identified one publication from an African setting that reported urinary concentrations of pesticides other than serum concentrations of DDT and other organochlorines [34]. This was a cross-sectional study that recruited 121 nonpregnant women working on farms during the pesticide spraying season in South Africa and a control group of 90 women living in nearby towns, and reported urinary concentrations of organophosphate and pyrethroid metabolites. Geometric means among both the farm workers and the townspeople in South Africa were higher for TCPy (farm: 6.15 µg/g; town: 4.14 µg/g) and 3-PBA (farm: 3.61 µg/g; town: 3.34 µg/g) than for our study participants, when comparing our creatinine-adjusted results. Differences in urinary concentrations may be related, at least in part, to pregnancy status, the sensitivity of the analytical methods used for the quantification of the pesticide biomarkers, or to the treatment of non-detectable concentrations in summary statistics.

### 4.2. Lessons Learned about the Conduct of Field Based Pesticide Research

There were two unexpected findings from our work. The lessons learned may benefit the design of future field-based research on pesticide exposure. First, in approximately one third of samples (16/51. 31.3%), creatinine concentrations were quite low (<27.2 mg/dL) for an adult population, despite being first morning voids. The lower values may be attributable, in part, to physiological changes in pregnancy, including an increased plasma volume, renal plasma flow, glomerular filtration rate, and filtration fraction, without any increase in the production of creatinine [35]. In this rural African population, it is also possible that these low urinary creatinine values reflect undernutrition and lower muscle mass, rather than hydration status. Therefore, in future studies in similar settings with pregnant women, we would suggest adjusting metabolite concentrations for specific gravity, rather than for creatinine.

Secondly, the frequent detection of specific organophosphate metabolites and parallel infrequent detection of nonspecific DAP metabolites was surprising. We speculate that these findings may be related to the stability of the compounds in urine; the specific organophosphate metabolites (PNP, TCPy, and IMPY) are eliminated as conjugates in urine, while DAPs are not. It is possible that storage conditions in an African field-based research setting had a higher impact on the integrity of urine for measuring DAPs compared to the other metabolites, if DAPs are more sensitive to temperature than the specific metabolites. Samples in our study were collected from participants’ homes in the community early in the morning on the day of collection and kept out of direct sunlight awaiting pickup. Samples were transported by motorbike in coolers with ice packs to the research center, before being frozen at −80 °C. Transportation time varied, ranging from 30 to 120 min, depending on the proximity of the community and the other research activities being performed by the research staff. As described above, the concentrations of the specific OP metabolites, TCPy and PNP, were more likely to be in the lower two quartiles for samples with undetectable concentrations of DAP metabolites. It seems plausible that the relatively low DAP metabolite concentrations may have decreased during transport to non-detectable concentrations by our method sensitivity. We must be careful not to draw strong conclusions given the limitations of the sample size. Nonetheless, it may be important to include specific OP metabolites, and not only rely on nonspecific DAPs, in future field-based research in Ghana or similar remote rural communities. Furthermore, direct comparisons with studies conducted in settings where samples are immediately frozen may be misleading, underestimating the exposure in certain field conditions.

### 4.3. Possible Sources of Pesticide Exposure

The sources of pesticide exposure in this primarily rural area of Ghana likely differ as compared with individuals in high resource settings. We acknowledge that our sample size is insufficient to establish exposure determinants and therefore only considered several factors for the purpose of a hypothesis-generating exploratory analysis.

The source of household water may affect pesticide exposure, as urinary concentrations of the organophosphate PNP, the pyrethroid 3-PBA, and the herbicide 2,4-D appear higher among women whose households used natural sources of water such as streams, lakes, or rivers, compared with households that sourced their water from pumps, pipes, or public taps. Occupational farming may be a predictor of high urinary 2,4-D concentrations, but did not appear predictive of organophosphate or pyrethroid insecticide biomarkers. It is possible that much of the food supply in the area is contaminated by pesticides, as suggested by a study of pesticide residues in the nearby city of Kumasi, which demonstrated that organophosphates were the major contributor, driving a combined consumer risk index above a threshold for concern [36]. Our questionnaire did not include information about the types of food eaten or the source of food supply; however, the food supply is predominantly locally grown in the study area, regardless of whether it is grown by the family or a neighbor. Exposure may not differ by personal or family farming practices, as essentially all of the individuals in our study catchment are eating locally produced foods. That said, the intake of fruits and vegetables may be different than in higher resource settings, impacting the overall exposure.

The ownership or utilization of bednets did not appear to correlate with urinary concentrations of the pyrethroid biomarker 3-PBA.

Our suggested predictors of exposure will need confirmation from larger studies. Of particular interest, we demonstrated rather low ICCs for the repeated samples for all of the analytes considered, with none above 0.35, such that approximately two-thirds or more of the variance was within-subject variability between samples. For PNP and 2,4-D, the ICC was <0.10 and, correspondingly, almost all of the variability was within a given study participant. This suggests that individual-level interventions have the potential for reducing exposure. Additionally, this emphasizes the need for repeated measures in epidemiological research in these settings.

### 4.4. Limitations and Future Directions

This pilot study recruited a relatively small number of women and obtained samples over a two-week period for each participant. In future work, measurements throughout the entire year would help inform whether seasonal agricultural spraying impacts exposure. A comparison to nonpregnant individuals would also be of value. We asked general questions about the residential and occupational use of pesticides, but did not review specific chemicals handled by the women in the day(s) prior to urine collection. In the parent cook stove trial, GRAPHS [18], from which we recruited for this pilot, a biobank of repeated urine collections was obtained over the course of pregnancy for our participants. We aim to leverage these resources in the future to more fully characterize exposure, routes of exposure, and connect exposure with the potential impact on pregnancy outcome and health in the first year of life. This is particularly important, as many of these chemical classes, such as organophosphates and potentially pyrethroids, have been associated with adverse neurodevelopment in children [1,2,37]. Assessing the health impacts of the herbicide 2,4 D during pregnancy may also be of interest, as it is being increasingly marketed in combination with glyphosate, both in resource poor and resource intensive environments.

## 5. Conclusions

This is one of the first studies conducted in a rural African population to document urinary concentrations of pesticide biomarkers. We recognize that the findings will need confirmation from larger studies, but our pilot work has demonstrated widespread exposure to select organophosphate insecticides and herbicides among pregnant women in this rural area of Ghana. For PNP and 2,4-D, concentrations were higher than those reported from a U.S. reference population of reproductive-aged women. In contrast, pyrethroid biomarker concentrations were lower than the U.S. reference and may reflect a lack of access to this class of compounds by rural Ghanaian households. Insecticide-treated bednet ownership and utilization did not appear to impact the exposure to pyrethroids. In an exploratory analysis, we identified the source of household water (natural vs. piped/pumped/tap) as a potential predictor of exposure to PNP and 2,4-D, or their precursors. Low ICCs for both of these biomarkers (0.07 and 0.06, respectively) suggest a high degree of within-person variability and the potential for individual level behavioral interventions to reduce exposure.

## Figures and Tables

**Table 1:** Cohort characteristics.

Demographics	*n* = 17
*Age* (years)	26.2 (±7.8)
*Gravidity*	3.4 (±2.8)
Education completed	
Primary school or less	10 (58.8%)
More than primary school	7 (41.2%)
*Religion*	
Catholic/Christian	10 (58.8%)
Muslim	3 (17.7%)
Atheist/Other	4 (23.5%)
*Occupation*	
Salaried (e.g., seamstress/hairdresser)	3 (17.6%)
Farmer/laborer/domestic worker	6 (35.3%)
None	8 (47.1%)
**Use of pesticides and bednets**	
*Use on farm or garden*	
Uses chemical(s) on farm/garden	14 (82.4%)
Type of chemical used on farm/garden *	
Insecticide	2 (14.3%)
Rodenticide	1 (7.1%)
Herbicide	14 (100%)
*Use in home*	
Type of chemical used in home	
Insecticide	6 (35.2%)
Rodenticide	7 (41.8%)
Location of pesticide use in home (if uses) **	
Bedroom	8 (100%)
Sitting room	2 (25.0%)
Kitchen	2 (25.0%)
Toilet/outhouse	0 (0%)
*Owns bednet*	13 (76.5%)
*Sleeps under bednet most nights*	11 (64.7%)

* Denominator = 14 for those reporting use of chemicals on farm or garden; ** Denominator = 8 for those reporting use of pesticides in and around the home.

**Table 2:** Urinary concentrations of biomarkers of organophosphate and pyrethroid insecticides, and select herbicides, among 17 pregnant women in rural Ghana (*n* = 51 samples).

Biomarker ^†^	Number > LOD (%)	LOD (µg/L)	Range of Detectable (µg/L)	GM in µg/L, (95% CI)	IQR (µg/L)	95th Centile (µg/L)	NHANES^28^ GM (µg/L) for Females 2009–2010 ^‡^	NHANES^28^ 95th Centile (µg/L) for Females 2009–2010 ^‡^	ICC
**OPs**									
*Specific*									
TCPy	40/51 (78.4%)	0.1	0.11–11.8	0.54 (0.36–0.81)	1.47	6.22	0.71 (0.63–0.79)	4.40 (4.09–4.73)	0.33
IMPY	19/49 (38.8%)	0.1	0.10–5.14	*	*	1.98	*	0.44 (0.31–0.60)	*
PNP	46/51 (90.1%)	0.1	0.19–8.26	0.71 (0.51–1.00)	1.34	4.65	0.40 (0.35–0.45)	3.07 (2.62–3.55)	0.07
*Nonspecific*									
DEDTP	2/51 (3.9%)	0.5	0.51–0.56	*	*	*	*	*	*
DEP	7/51 (13.7%)	0.1	0.58–14.90	*	*	*	*	14.5 (10.7–18.8)	*
DMDTP	9/51 (17.6%)	0.1	0.11–5.16	*	*	*	*	5.54 (4.06–6.69)	*
DMTP	14/51 (27.4%)	0.1	0.15–8.50	*	*	2.15	2.24 (1.88–2.66)	37.7 (27.0–50.1)	*
DETP	6/51 (11.8%)	1	1.05–3.06	*	*	*	*	4.01 (2.41–6.59)	*
**Pyrethroids**									
4-F-3-PBA	2/51 (3.9%)	0.1	0.16–0.67	*	*	*	*	*	*
3-PBA	37/49 (75.5%)	0.1	0.11–9.70	0.23 (0.17–0.32)	0.27	1.38	0.42 (0.37–0.47)	6.5 (4.89–8.50)	0.31
*Trans*-DCCA	2/51 (3.9%)	0.6	1.99–3.15	*	*	*	*	5.51 (2.87–9.82)	*
**Herbicides**									
2,4-D	36/51 (70.6%)	0.15	0.15–166.0	0.46 (0.29–0.73)	0.89	17.4	0.28 (0.25–0.30)	1.1 (0.97–1.39)	0.06
2,4,5-T	0/51 (0%)	0.1	*	*	*	*	*	*	*

CI = confidence interval; Cr = creatinine; GM = geometric mean; ICC = intraclass correlation coefficient; IQR = interquartile range; LOD = limit of detection; NHANES = National Health and Nutrition Examination Survey; OPs = organophosphates. ^†^ Parent chemicals of the measured analytes: 3,5,6-Trichloro-2-pyridinol (TCPy) = chlorpyrifos, chlorpyrifos-methyl; 2-isopropyl-4-methyl-6-hydroxypyrimidine (IMPY) = diazinon; *para*-Nitrophenol (PNP) = parathion, methyl parathion; 4-fluoro-3-phenoxybenzoic acid (4-F-3-PBA) = cyfluthrin; 3-phenoxybenzoic acid (3-PBA) = cyhalothrin, cypermethrin, deltamethrin, fenpropathrin, permethrin, tralomethrin; trans-3-2,2-Dichlorovinyl-2,2-dimethylcyclopropane carboxylic acid (*trans*-DCCA) = permethrin, cypermethrin; cyfluthrin; 2,4-Dichlorophenoxyacetic acid (2,4-D) = 2,4-Dichlorophenoxyacetic acid and its esters; 2,4,5-Trichlorophenoxyacetic acid (2,4,5-T) = 2,4,5-Trichlorophenoxyacetic acid; * Not calculated: proportion of results below limit of detection was too high to provide a valid result; ^‡^ Most recent data from NHANES for the nonspecific organophosphates (dialkyl phosphate metabolites) is from years 2007–2008.

## Supplementary Materials

The following are available online at www.mdpi.com/1660-4601/14/4/354/s1, Table S1: Creatinine-adjusted urinary concentrations of biomarkers of organophosphate and pyrethroid insecticides, and select herbicides among 17 pregnant women in rural Ghana (*n* = 51 samples); Table S2: Potential determinants of pesticide and herbicide urinary concentrations among study participants.
