# Supplementary material for: Urinary Concentrations of Insecticide and Herbicide Metabolites among Pregnant Women in Rural Ghana: A Pilot Study
Source: Int J Environ Res Public Health. 2017 Mar 29;14(4):354. doi: 10.3390/ijerph14040354 (PMC5409555; doi:10.3390/ijerph14040354)
Supplement: Supplementary file 1 [file ijerph-14-00354-s001.pdf]

Supplementary Materials: Urinary Concentrations of Insecticide and Herbicide Metabolites among Pregnant Women in Rural Ghana: A Pilot Study

Table S1: Creatinine-adjusted urinary concentrations of biomarkers of organophosphate and pyrethroid insecticides, and select herbicides among 17 pregnant women in rural Ghana (n=51 samples).

Biomarker†	Number > LOD (%)	LOD (µg/L)	Range of detectable (µg/g)	GM in µg/g, Cr-corrected (95% CI)	IQR (µg/g)	95th centile (µg/g)	NHANES[28] GM (µg/g) for females 2009-10ǂ	NHANES[28] 95th centile (µg/g) for females 2009-10ǂ	ICC
OPs									
Specific									
TCPy	40/51(78.4%)	0.1	0.50-13.36	1.32 (0.92-1.89)	2.45	9.15	0.86 (0.77-0.95)	3.68 (3.18-4.10)	0.41
IMPY	19/49(38.8%)	0.1	0.12-5.48	*	*	1.83	*	0.58 (0.50-0.71)	*
PNP	46/51 (90.1%)	0.1	0.50-16.63	1.73 (1.34-2.24)	1.81	10.20	0.48 (0.43-0.54)	2.72 (2.32-3.46)	0.42
Nonspecific									
DEDTP	2/51 (3.9%)	0.5	0.84-6.91	*	*	*	*	*	*
DEP	7/51 (13.7%)	0.1	0.45-37.77	*	*	*	*	16.5 (13.1-20.9)	*
DMDTP	9/51 (17.6%)	0.1	0.15-5.11	*	*	*	*	7.18 (4.22-11.1)	*
DMTP	14/51(27.4%)	0.1	0.18-8.42	*	*	4.21	2.81 (2.40-3.29)	41.2 (27.4-60.8)	*
DETP	6/51 (11.8%)	1	0.60-7.76	*	*	*	*	4.48 (3.30-5.73)	*
Pyrethroids									
4-F-3-PBA	2/51 (3.9%)	0.1	0.29-1.62	*	*	*	*	*	*
3-PBA	37/49 (75.5%)	0.1	0.13-9.61	0.58 (0.43-0.77)	0.85	3.71	0.51 (0.45-0.56)	5.86 (4.58-8.90)	0.03
Trans-DCCA	2/51 (3.9%)	0.6	3.12-7.32	*	*	*	*	5.30 (3.54-8.27)	*
Herbicides									
2,4-D	36/51 (70.6%)	0.15	0.19- 715.83	1.11 (0.70-1.76)	1.96	44.80	0.33 (0.30-0.37)	1.40 (1.21-1.55)	0.02
2,4,5-T	0/51 (0%)	0.1	Not applicable	*	*	*	*	*	*

CI= confidence interval; Cr= creatinine; GM= geometric mean; ICC= intraclass correlation coefficient; IQR= interquartile range; LOD= limit of detection; NHANES= National Health and Nutrition examination Survey; OPs= organophosphates.

* Not calculated: proportion of results below limit of detection was too high to provide a valid result.

†Parent chemicals of the measured analytes: 3,5,6-Trichloro-2-pyridinol (TCPy)= chlorpyrifos, chlorpyrifos-methyl; 2-isopropyl-4-methyl-6-hydroxypyrimidine (IMPY)= diazinon; *para*-Nitrophenol (PNP)= parathion, methyl parathion; 4-fluoro-3-phenoxybenzoic acid (4-F-3-PBA)= cyfluthrin; 3-phenoxybenzoic acid (3-PBA)=cyhalothrin, cypermethrin, deltamethrin, fenpropathrin, permethrin, tralomethrin; trans-3-2,2-Dichlorovinyl-2,2-dimethylcyclopropane carboxylic acid (*trans*-DCCA)= permethrin, cypermethrin; cyfluthrin; 2,4-Dichlorophenoxyacetic acid (2,4-D)= 2,4-Dichlorophenoxyacetic acid and its esters; 2,4,5-Trichlorophenoxyacetic acid (2,4,5-T)= 2,4,5-Trichlorophenoxyacetic acid.

ǂ Most recent data from NHANES for the nonspecific organophosphates (dialkyl phosphate metabolites) is from years 2007-08.

Table S2: Potential determinants of pesticide and herbicide urinary concentrations among study participants.

	Organophosphates GM in µg/L (n)		Pyrethroids GM in µg/L	Herbicide GM in µg/L
	TCPy	*PNP*	*3-PBA*	*2,4-D*
Age				
≥ 30 years (n=5)	0.43	0.71	0.36	1.48
< 30 years (n=12)	0.59	0.71	0.19	0.28
Education				
Primary school or less (n=10)	0.37	0.73	0.25	0.66
More than primary school (n=7)	0.93	0.68	0.20	0.27
Farming				
Is farming your occupation?				
Yes (n=6)	0.27	0.63	0.21	0.95
No (n=11)	0.80	0.76	0.24	0.31
Residential use of pesticides				
Does the household use chemicals to kill insects/rodent in or around the home?				
Yes (n=8)	0.50	0.66	0.21	0.37
No (n=9)	0.58	0.76	0.25	0.55
Source of water				
What is the household source of water?				
Pumped, piped or public tap (n=13)	0.57	0.65	0.18	0.31
From natural stream/river/lake (n=4)	0.45	0.96	0.53	1.57
Malaria control measures				
Household owns bednet?				
Yes (n=13)	--	--	0.24	--
No (n=4)	--	--	0.21	--
Slept under a bed last night?				
Yes (n=10)	--	--	0.26	--
No (n=7)	--	--	0.19	--
Sleeps under bednet most nights?				
Yes (n=11)	--	--	0.25	--
No (n=6)	--	--	0.20	--

2,4-D= 2,4-Dichlorophenoxyacetic acid; 3-PBA= 3-phenoxybenzoic acid; Cr= creatinine; GM= geometric mean; IMPY=2-isopropyl-4-methyl-6-hydroxypyrimidine; PNP= *para*-Nitrophenol; TCPy=3,5,6-Trichloro-2-pyridinol.
